# SalmoBase: an integrated molecular data resource for *Salmonid* species

**DOI:** 10.1186/s12864-017-3877-1

**Published:** 2017-06-26

**Authors:** Jeevan Karloss Antony Samy, Teshome Dagne Mulugeta, Torfinn Nome, Simen Rød Sandve, Fabian Grammes, Matthew Peter Kent, Sigbjørn Lien, Dag Inge Våge

**Affiliations:** Centre for Integrative Genetics (CIGENE), Department of Animal and Aquacultural Sciences (IHA), Faculty of Biosciences (BIOVIT), Norwegian University of Life Sciences (NMBU), 1432 Ås, Akershus, Norway

**Keywords:** Salmobase, Atlantic salmon, Salmonids, Genome browser

## Abstract

**Background:**

Salmonids are ray-finned fishes which constitute 11 genera and at least 70 species including Atlantic salmon, whitefishes, graylings, rainbow trout, and char. The common ancestor of all Salmonidae experienced a whole genome duplication (WGD) ~80 million years ago, resulting in an autotetraploid genome. Genomic rediplodization is still going on in salmonid species, providing an unique system for studying evolutionary consequences of whole genome duplication. In recent years, high quality genome sequences of Atlantic salmon and Rainbow trout has been established, due to their scientific and commercial values. In this paper we introduce SalmoBase (http://www.salmobase.org/), a tool for making molecular resources for salmonids public available in a framework of visualizations and analytic tools.

**Results:**

SalmoBase has been developed as a part of the ELIXIR.NO project. Currently, SalmoBase contains molecular resources for Atlantic salmon and Rainbow trout. Data can be accessed through BLAST, Genome Browser (GBrowse), Genetic Variation Browser (GVBrowse) and Gene Expression Browser (GEBrowse).

**Conclusions:**

To the best of our knowledge, SalmoBase is the first database which integrates salmonids data and allow users to study salmonids in an integrated framework. The database and its tools (e.g., comparative genomics tools, synteny browsers) will be expanded as additional public resources describing other Salmonidae genomes become available.

## Background

Salmonids (e.g. Atlantic salmon (*Salmo salar*), Rainbow trout (*Oncorhynchus mykiss*), Brown trout (Salmo trutta)) has considerable socio- and economic importance. From a biological perspective the anadromous migration pattern of salmon is of great interest, and allow investigations of unique physiological traits such as smoltification and flesh pigmentation. The evolutionary history of salmonids is particularly interesting. A whole genome duplication (WDG) event took place in a common ancestor to all salmonids ~80 million years ago [1], which makes it possible to study post duplication phenomena in a recent time frame, in contrast to other polyploid origin vertebrates whose WGDs date back further in time. These phenomena include the effects of WGDs on gene diversity and functional specialization, as well as consequences on evolution and adaptation [2].

A high quality, annotated Atlantic salmon and Rainbow trout genome sequences are now available thanks to the efforts from the International Cooperation to Sequence the Atlantic Salmon (ICSASG) and associated partners [3] and The international collaboration to sequence Rainbow trout genome, and we expect that genome sequences and genomic data for other salmonid species will be available in the near future. SalmoBase (www.salmobase.org) was developed to make these substantial amounts of data accessible through visualizations and analytic tools in a common framework. We expect that genome sequences and genomic data for other salmonid species will be available in the near future and plan to integrate this information with SalmoBase.

As a first step, the genome and genome annotations for Atlantic salmon and Rainbow trout are made available through SalmoBase. For Atlantic salmon, tissue specific gene expression data and single nucleotide polymorphisms (SNP) data are also available. Similar resources for other salmonid species will be added to SalmoBase when they become available.

## Construction and content

SalmoBase was developed using HTML, PHP, Javascript, Python and MySQL. The latest version of GBrowse [4] and BLAST [5] are installed. Gene expression data are plotted using Plotly (plot.ly) Javascript.

Atlantic salmon genome reference (fasta file), annotation (gff3 file) and gene expression data (Sequence Read Archive accession: PRJNA260929) were produced as the part of ICSASG. The RefSeq annotation for Atlantic salmon was added later when it became available. New Rainbow trout genome reference (fasta file) and annotations (gff3) were produced by The international collaboration to sequence the Rainbow trout genome.

## Utility and discussion

### Genome browser (GBrowse)

**Fig. 1:**
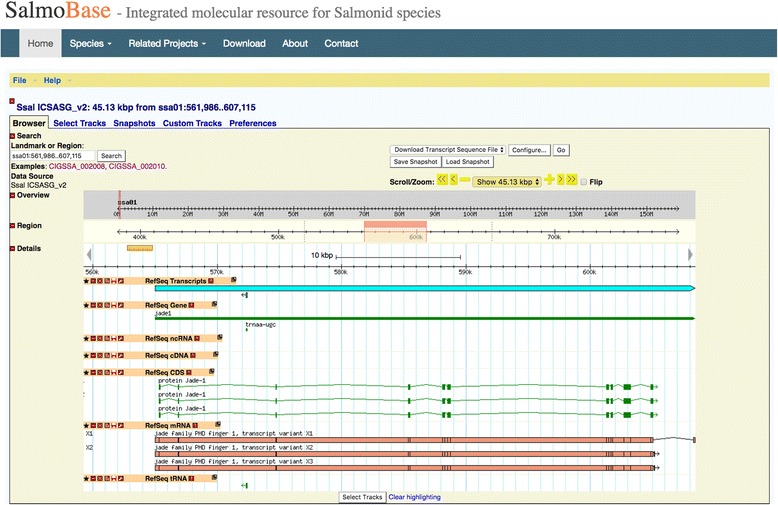
Atlantic salmon genome browser contains two sets of Atlantic salmon genome annotations based on an in-house annotation pipeline and the NCBI annotation pipeline

We have chosen to use GBrowse [4] to visualize the genomic data for salmonids. Atlantic salmon [3] GBrowse contains two sets of genome annotations (Fig. 1), one made in-house during the assembly of the salmon genome and the other presenting the NCBI RefSeq annotation which, providing an option to compare results between the two different annotations. Currently, the Atlantic salmon genome browser contains genes, transcripts and repeat sequences. The transcript tracks are linked to tissue specific gene expression data, homeolog regions and sequence download options. Rainbow trout GBrowse contains the latest reference genome and an in-house genome annotation.

Data tracks can be downloaded in a variety of formats including GFF3, Genbank, and EMBL (European Molecular Biology Laboratory), while gene, protein and transcript sequences can be downloaded in FASTA, Genbank, and EMBL formats etc. Users can upload their own data (custom track option) in a variety of file formats and can customize track displays to visualize their data. Users can easily save and share search results as links, or export the results as PNG, SVG and other file formats for publication purposes. Navigation in the database is eased by clickable questionmarks.

### BLAST server

**Fig. 2:**
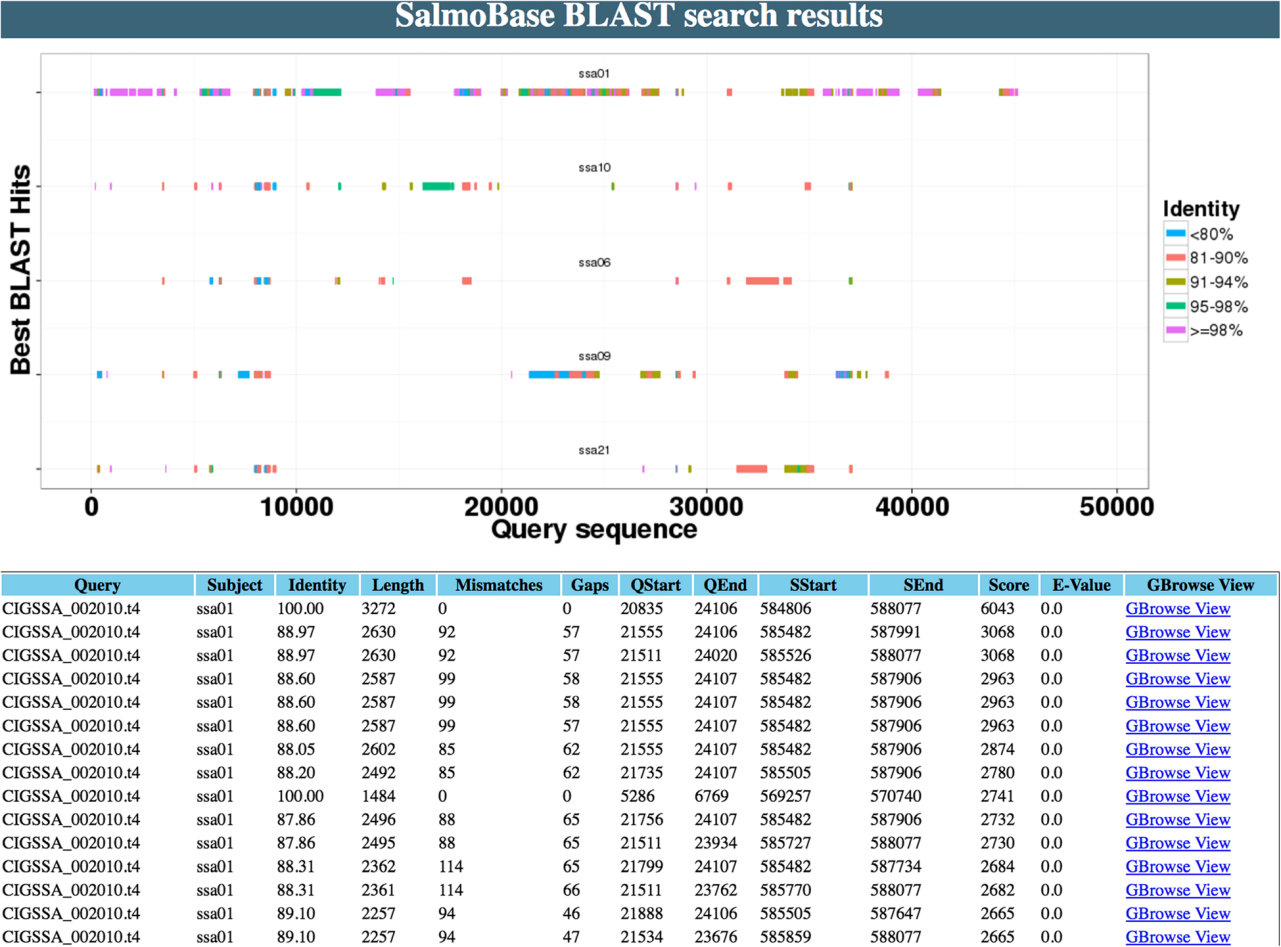
BLAST search allows users to search for homologous regions in the entire reference genome, repeat masked genome, predicted protein sequences (CIGENE and/or RefSeq), and transcript sequence databases

SalmoBase BLAST [5] search allows the users to search their sequences against the entire reference genome, repeat masked genome, predicted protein sequences (CIGENE and/or RefSeq), as well as transcript sequence databases. The five top hits from BLAST are displayed including location of the alignment, size of the alignment and similarity between query and subject (Fig. 2). Search results are connected to the GBrowse so that information about nearby genes and other genomic features can be accessed.

### Genetic variation browser (GVBrowse)

**Fig. 3:**
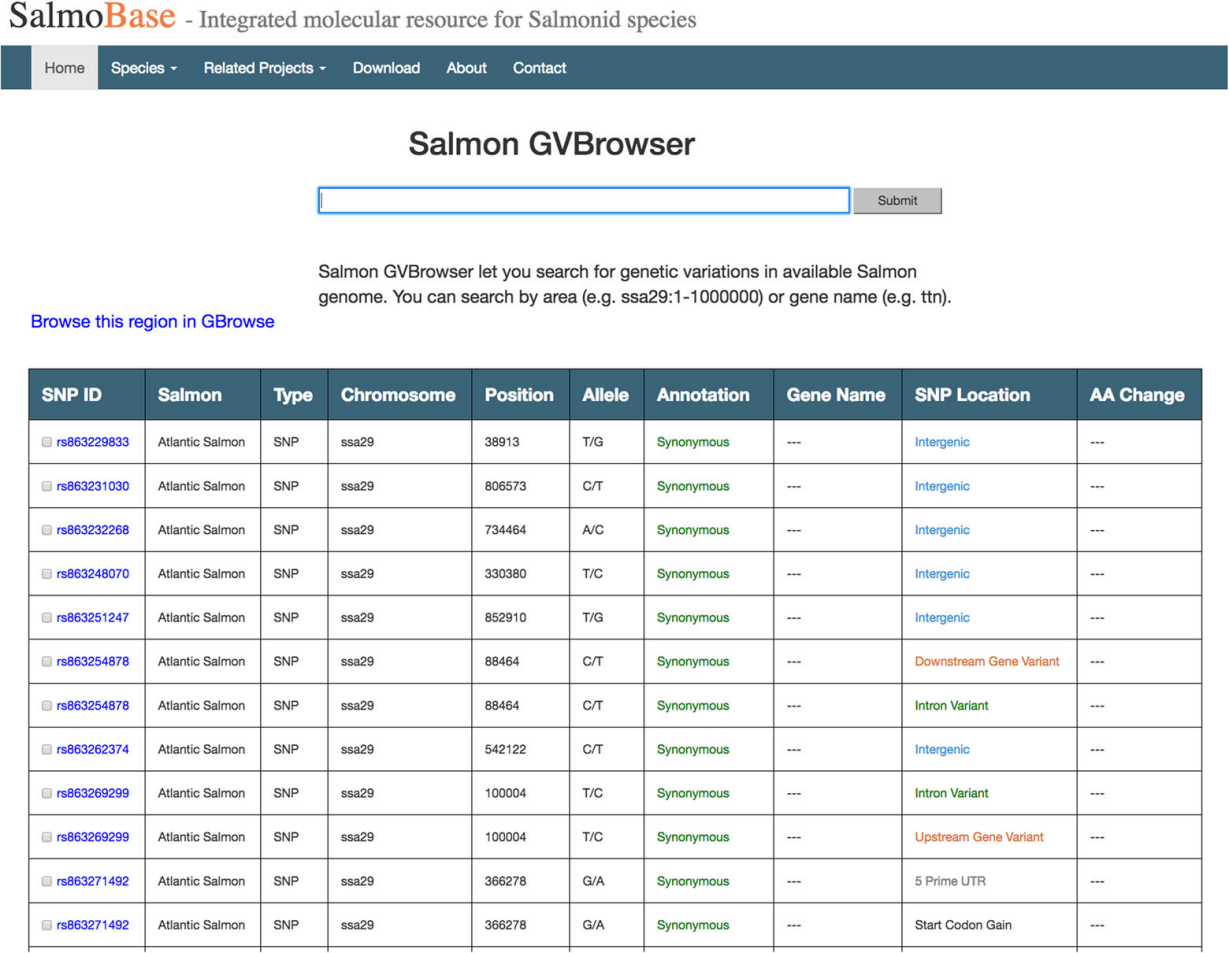
Genetic variation browser (GVBrowser) lists publically available genetic variation data in table format for Atlantic salmon based on the search parameters

A genetic variation browser displays genetic variations in the Atlantic salmon genome. Genetic variations can be shown based on genomic location (e.g., ssa01:1–1000) or by gene symbols (e.g., ttn) (Fig. 3). Resulting SNPs and other DNA variations are displayed in a table format along with location, alleles, annotation (synonymous or non-synonymous), and location with respect to genes (intron, exon, upstream, downstream, or inter-genic).

It is possible to quickly obtain flanking sequence for each variation by following the link from the “SNP ID”. By clicking the genomic view image in the SNP ID link, additional information can also be obtained such as location of the genetic variation in genome sequence, nearby gene annotations and other genomic features. Flanking sequences for multiple genetic variations can be downloaded by selecting the wanted genetic variations and clicking the download button at the bottom of the result table.

### Gene expression browser (GEBrowse)

**Fig. 4:**
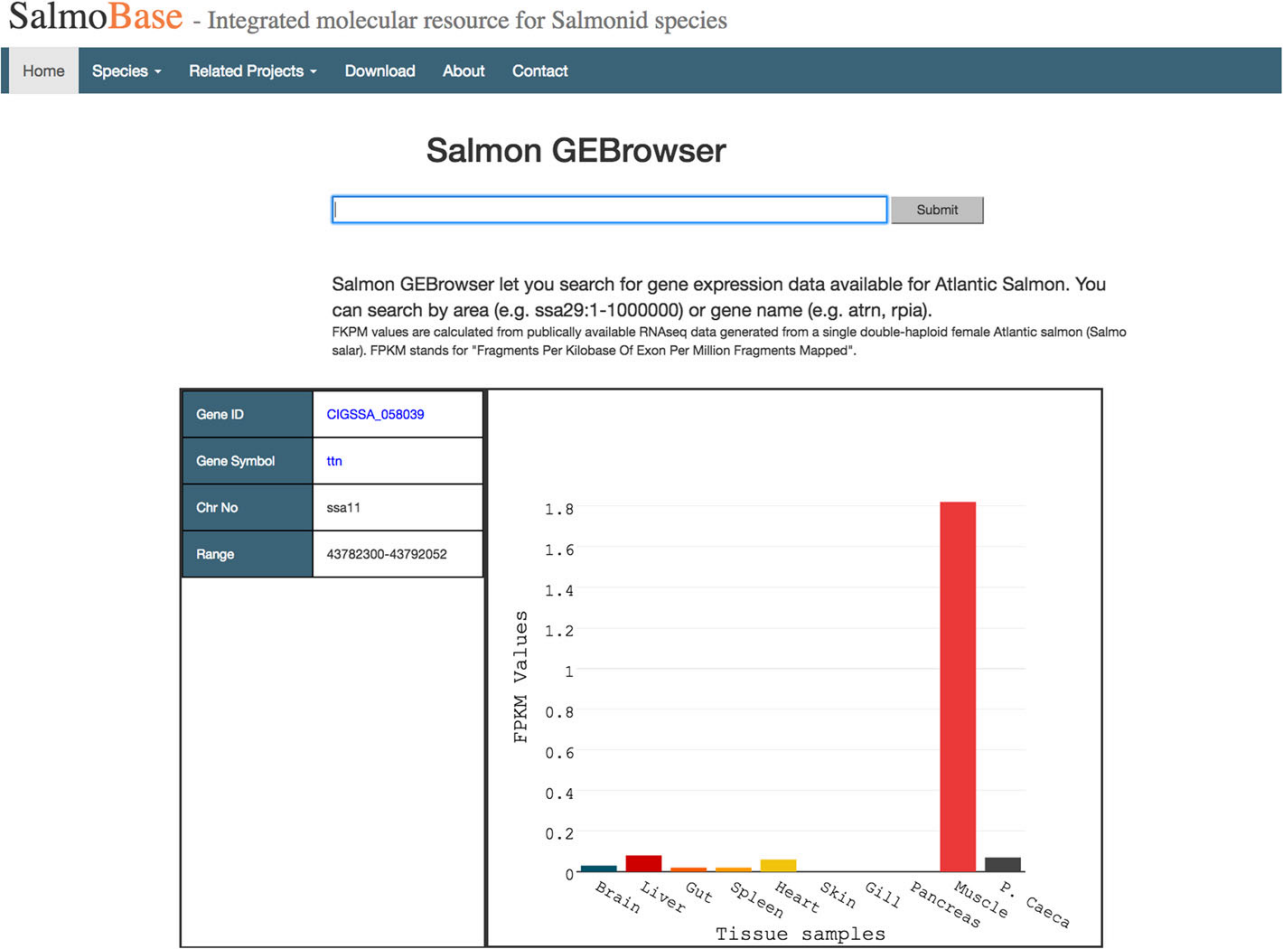
Gene expression browser (GEBrowser) displays publically available tissue specific gene expression data as bar graphs based on the search parameters

The gene expression browser allows the users to access the tissue-specific gene expression data of Atlantic salmon. Users can search the gene expression browser by genomic coordinates (e.g., ssa02:1–100,000) or by gene symbols (e.g., ttn). Pre-plotted bar graphs (Fig. 4) based on the tissue-specific Fragments per Kilobase of Exon per Million Fragments Mapped (FPKM) values are displayed.

### Future plans and intergration of other resources

SalmoBase was developed in close collaboration with ICSASG research groups. Through this collaboration more resources from other projects on salmonids will become available in the near future. As more data becomes available for Atlantic salmon, Rainbow trout and other salmonid species, new tools and resources will be added to SalmoBase. The SalmoBase team is also working closely with Functional Analysis of All Salmonid Genomes (FAASG) [6] and the results from FAASG will be accessible through Salmobase in the future.

### Database access and feedback

Data are available for download under the ‘Download’ option in SalmoBase. User support is available through the ‘Contact’ form in SalmoBase. Suggestions for improvements and other comments are welcomed through the ‘Contact’ form. We will consider to include data from users who wish to deposit data into SalmoBase.

## Conclusions

To the best of our knowledge SalmoBase is the only online database to access, visualize and download genomics data of salmonids. Due to rapid improvements in high-throughput sequencing technologies we expect a deluge for salmonids’ genomics data. SalmoBase is designed to accommodate the challenges. And, SalmoBase will play a vital role in studying salmonids.

## Availability and requirements

SalmoBase can be accessed at www.salmobase.org.

## Abbreviations

EMBL: European Molecular Biology Laboratory
FAASG: Functional Analysis of All Salmonid Genomes
FPKM: Fragments per Kilobase of Exon per Million Fragments Mapped
GBrowse: Genome Browser
GEBrowse: Gene Expression Browser
GVBrowse: Genetic Variation Browser
ICSASG: International Cooperation to Sequence the Atlantic Salmon
SNP: single nucleotide polymorphisms
WGD: Whole Genome Duplicatio
